# Fifteen-year trajectories of multimorbidity and polypharmacy in Dutch primary care—A longitudinal analysis of age and sex patterns

**DOI:** 10.1371/journal.pone.0264343

**Published:** 2022-02-25

**Authors:** Rein Vos, Jos Boesten, Marjan van den Akker

**Affiliations:** 1 Department of Methodology and Statistics, Maastricht University, Maastricht, The Netherlands; 2 Department of Family Medicine, Care and Public Health Research Institute, Maastricht University, Maastricht, The Netherlands; 3 Institute of General Practice, Johann Wolfgang Goethe University, Frankfurt am Main, Germany; 4 Department of Public Health and Primary Care, Academic Centre of General Practice, KU Leuven, Leuven, Belgium; University of Manitoba, CANADA

## Abstract

**Objective:**

After stratifying for age, sex and multimorbidity at baseline, our aim is to analyse time trends in incident multimorbidity and polypharmacy in the 15-year clinical trajectories of individual patients in a family medicine setting.

**Methods:**

This study was carried out using data from the Registration Network Family Medicine in the South of the Netherlands. The clinical trajectories of 10037 subjects during the 15-year period (2000–2014) were analyzed in a repeated measurement of using a generalized estimating equations model as well as a multilevel random intercept model with repeated measurements to determine patterns of incident multimorbidity and polypharmacy. Hierarchical age-period-cohort models were used to generate age and cohort trajectories for comparison with prevalence trends in multimorbidity literature.

**Results:**

Multimorbidity was more common in females than in males throughout the duration of the 15-year trajectory (females: 39.6%; males: 33.5%). With respective ratios of 11.7 and 5.9 between the end and the beginning of the 15-year period, the youngest female and male groups showed a substantial increase in multimorbidity prevalence. Ratios in the oldest female and male groups were 2.2 and 1.9 respectively. Females had higher levels of multimorbidity than males in the 0-24-year and 25-44-year age groups, but the levels converged to a prevalence of 92.2% in the oldest male and 90.7% in the oldest female group. Similar, albeit, moderate differences were found in polypharmacy patterns.

**Conclusions:**

We sought to specify the progression of multimorbidity from an early age. As a result, our study adds to the multimorbidity literature by specifying changes in chronic disease accumulation with relation to polypharmacy, and by tracking differences in patient trajectories according to age and sex. Multimorbidity and polypharmacy are common and their prevalence is accelerating, with a relatively rapid increase in younger groups. From the point of view of family medicine, this underlines the need for a longitudinal approach and a life course perspective in patient care.

## Introduction

Multimorbidity—the co-occurrence of two or more chronic health conditions in a patient [1]—and polypharmacy—the prescription of five or more different medications in one year [2]—are broadly recognized as challenges facing patients, health care professionals and society at large [3]. The prevalence of multimorbidity in the general population is 20–30% [4]. Patients with multimorbidity account for around 80% of all consultations with general practitioners and virtually all consultations with geriatricians. They also require substantial health care resources and complex pharmacotherapeutic regimes [5]. There is clear evidence that multimorbidity negatively influences the quality of life of patients [5, 6].

According to studies, multimorbidity affects between 55 to 98% of the older population, depending on definition, age of the population and the data source [7, 8]. The proportion of patients with multimorbidity increases strongly with age, with the proportion of females being statistically significantly higher in all age groups. Although prevalence increases substantially with age, in absolute terms multimorbidity is much more common in those aged 65 years or less [8, 9]. Some studies have indicated interactive associations between sex, age and multimorbidity, which may depend on medical, lifestyle and social factors influencing the health status of patients [8, 10]. Data on the age at which multimorbidity begins and how it evolves over time are urgently needed in order to develop focused interventions that help prevent multimorbidity and associated adverse health outcomes [11].

As with multimorbidity, the prevalence of polypharmacy among persons aged 65 and older has increased significantly over the past 20 years, with recent international estimates ranging between 50–66% [7, 10]. Multimorbidity and polypharmacy are closely related. Polypharmacy is frequently found among patients with multimorbidity, and the number of their chronic conditions is an even stronger predictor of the number of prescribed medications they are taking than age is [7]. However, the relationship between multimorbidity and polypharmacy is complex [3], and the interaction between them is both synergistic and antagonistic [1, 7, 10, 12, 13]. Maxwell et al. (2021) note that recent studies have explored broad trends in multimorbidity and/or polypharmacy but few have examined sex- and age-specific trends, or investigated the impact of multimorbidity and age on medication use over time [10].

Many studies suggest that chronic health conditions and multimorbidity are increasingly common [1, 9, 14], but discrepancies exist in reported findings. In addition to methodological differences between studies, an important explanation, noted by Vetrano et al. (2020), lies in the dynamic nature of disease courses, which is not accounted for in cross-sectional studies [5]. Indeed, available information on patterns in the prevalence of multimorbidity and polypharmacy is mainly based on cross-sectional studies at different times and in different populations [1, 5, 9]. Studies on clinical trajectories at an individual patient level are very limited, and generally focus on specific conditions such as dementia, atopic condition, depression or biological and lifestyle factors, and health care costs and resources [15–18]. Furthermore, most studies focus on older adults [10, 19–21], while the study of childhood multimorbidity is still in its infancy [22, 23]. In a recent systematic review Kudesia et al. summarized the 32 studies that contained information on incident multimorbidity [24]. Of these, 12 were based on medical records, while the others were based on self-report data, and all of them either reported cumulative incidence (N = 18) or incidence (N = 14). All studies defined incident multimorbidity as the development of the second of a list of chronic conditions over a specific period. Across studies, incidence rates ranged from 1.26 to 342 per 1000 patient-years [24]. Ten studies reported unadjusted cumulative incidence rates ranging from 1.3% to 61.0%. Most studies did not stratify incidence rates by sex and only two studies adjusted incidence for age and sex [24]. Incidence rates increased with age, with studies of middle-aged to older participants (50 years of age or older) reporting higher incidence rates than those consisting of younger and adult populations (0–40 years of age). Only one study calculated incidence rates for age and sex groups separately over a 14-year period, but it focused on combinations of 20 conditions (11). Most studies limited the number of reviewed chronic conditions to between 3 and 30, apart from five studies, which included between around one hundred and several hundred chronic conditions [24]. Only four studies included younger subjects aged under 20 years, of which two were confined to medulloblastoma survivors and patients with asthma, rhinitis and other respiratory disorders [11, 24, 25]. Many studies focused only on the incidence rate of chronic conditions in specific age groups over a follow-up period of 3–5 years. Some studies used a longer time period of 15 to 25 years, and made use of follow-up clinical assessments or patient questionnaires taking place every 4–5 years, whereby the research question was whether the prevalence of multimorbidity increased in a specific age group over time [11, 24–26]. Only few studies used a trajectory approach and then with a limited set of 7–30 chronic conditions [11, 23]. None of the studies investigated the relationship between incident multimorbidity and medication prescriptions and polypharmacy. Furthermore, few polypharmacy studies investigated longitudinal trajectories in polypharmacy [10, 27, 28], and, if performed, mostly in older age [29–33].

Vetrano noted that rather than to analyze groups of individuals, most previous studies in this field focused on multimorbidity from the viewpoint of diseases [5]. The authors of that study therefore aimed to identify multimorbidity clusters in a population-based cohort of adults and to follow the clinical trajectories of the individuals as they moved between the clusters over time. We followed a similar track, but did choose a family medicine based cohort of young and adult persons, whereby we used a discrete time-to-event method to model the clinical trajectories of the individuals in terms of the incidence of multimorbidity and its relationship with polypharmacy after stratifying age, sex and the presence or absence of multimorbidity at baseline.

## Materials and methods

### Data source and setting

This study was carried out using data from the Research Network Family Medicine (RNFM [34]), which collects information from general practitioners (GPs) working in family medicine practices in the South of the Netherlands. The RNFM database contains data from around 80,000 active patients. All relevant health problems are registered. A health problem is defined as ‘anything that has required, does or may require health care management and has affected or could significantly affect a person’s physical or emotional well-being’ [18]. Participating GPs systematically register all health problems, which are coded according to the International Classification of Primary Care (ICPC) and the criteria of the International Classification of Health Problems in Primary Care (ICHPPC-2) [18, 35]. ICPC is a diagnostic classification method that was developed under the umbrella of WONCA and the WHO and is based on other diagnostic classifications such as the ICD [18, 35]. When medical conditions are complex, registration is almost always based on a specialist diagnosis reported to the GP. In the Netherlands, GPs have comprehensive information on the health status of their patients because GPs function as gatekeepers to specialized health care facilities. All Dutch residents must have health care insurance and register with a GP. GPs only enter health problems that fulfil ICHPPC criteria into the general database. Every three months the GP transfers the coded health problems to the RNFM database. When patients are newly enrolled in a practice, significant morbidity is retrospectively entered into their electronic medical records, thus providing a comprehensive view of the health status of patients throughout their lives. Membership of the RNFM population ends with migration or death. All patients included in the RNFM database have been informed about the anonymous use of their health information and may opt out of the system if they wish. The quality of the data is safeguarded through the use of instruction and training sessions, regular regional consensus groups, quality control audits, the availability of an online thesaurus when entering data, and systematic control software [36, 37]. Participating GPs are trained in the structured and automated registration of health problems by means of ICPC codes, and have additional computer facilities for registration and coding. The socio-demographic characteristics of the RNFM population are comparable to those of the overall Dutch population. The RNFM is thus a valid and precise database for medical research.

The RNFM database is embedded in a family practice network in the South of the Netherlands, which covers an open and dynamic population. The identification data of the patient are checked individually and controlled both at the family practice level and at the RNFM-network level through the use of various checking algorithms. The number of people opting out is very low, numbering only a few patients per practice or below 0.5% of the population. A reference group of 22 practices and 77 general practitioners also participate by continuously controlling and checking the data. Special attention is paid to the stability of the entire system over the years. The three family medicine practices included in this study have been members of the RNFM for a long period of time and more importantly, their data belong to a dataset that has been used in previous research, controlled and checked by medically trained investigators, and linked with prescription data [18, 35]. Data cleaning also yielded an error rate of below 0.5% [18, 35]. As family practices may exhibit differences in the composition of their patients in terms of relevant medical and demographic variables, the RNFM networks conducts internal validation comparisons. External validation employs national and international comparisons. Nationally, RNFM data were compared with the Tweede nationale studie (the Second Dutch national survey of general practice), that studied morbidity and care in Dutch general practice [38]. In this paper, comparisons were made between the prevalence of chronic health conditions and multimorbidity in the study population, and the general patient population included in the RNFM database.

Medication prescribed in general practice is also entered into the system using ATC codes (Anatomical Therapeutic Chemical codes) [39]. Completeness and reliability are assured through the use of digitalized medication prescriptions. Medication prescriptions from medical specialists are not automatically included. However, in the Netherlands repeated prescriptions are usually provided by the GP, so the data is included with only a short delay. Internal validation is performed using so-called tracers, i.e., medications which are supposedly linked to a specific medical condition [40]. For example, for diabetes and anti-diabetics, 88% of the people who were prescribed anti-diabetics (ATC: A10A, A10B and A10X) also had a diabetes diagnosis. International comparison between the RNFM and the Flemish-Belgian INTEGO databases showed satisfactory results [40].

### Design and study population

The practice population represented in the RNFM database was used for the repeated measurement of multimorbidity and polypharmacy patterns during a 15-year period. The closed cohort of 10037 persons was followed from January 1, 2000 to December 31, 2014. The 10037 individuals were selected from three family practices within RNFM that were participating centers, had a steady patient population in the period under review, and that provided links to the registration of health problems and prescribed medications at an individual patient level throughout the observation period; the data used were selected from a dataset used in a previous study [35]. The prevalence patterns and trends in the prescribed medications and chronic health problems of this subset of patients were compared with the patterns and trends in the overall RNFM database. The RNFM data also include a limited number of demographic and medical variables such as age and sex.

Only health problems that were *chronic*, *permanent* or *recurrent* health problems [18, 35] that were recorded by the GP and were expected to have lasting consequences for the functional status or prognosis of the patient, were selected from the RNFM database. Chronic problems were defined as health problems lasting longer than six months, permanent problems are defined as health problems from which no recovery could be expected), and recurrent problems as health problems that had recurred three times or more over a period of six months. We used the ICPC-1 version of the codes of chronic health conditions as defined by the Belgian INTEGO-network of family medicine practices [41, 42], which contains 88 chronic health conditions (see S1 Table). All sixteen ICPC Chapters containing diagnostic codes related to chronic health problems were included: A (General), B (Blood, D (Digestive), F (Eye), H (Ear), K (Circulatory), L (Locomotor), N (Neurological), P (Psychiatric, psychological), R (Respiratory), S (Skin), T (Endocrine, metabolic and nutritional), U (Urinary conditions), W (Pregnancy related health problems), X (Female conditions), Y (Male conditions).

### Measures

For this study, multimorbidity was defined as the co-occurrence of two or more chronic diseases in an individual. Persons with two or more of the 88 chronic conditions at baseline were considered to have prevalent multimorbidity which we indicated by using a nominal variable to denote the presence or absence of multimorbidity. The incidence of each of the 88 chronic conditions was assessed for the persons free of those conditions at baseline. Incidence rates were calculated separately by age (using four age strata), sex and the presence or absence of multimorbidity at baseline.

For the evolution of multimorbidity the yearly incidence of new chronic health conditions, or incident morbidity was used to measure the evolution of multimorbidity, while incidence proportion—or cumulative incidence, also expressed as percentage—was used to measure the proportion of subjects who developed a state of multimorbidity during a specified period [43]. Thus, we used event times and intervals for a closed population over a 15-year period [43], which can be interpreted as a discrete time-to-event approach [44]. Similarly, polypharmacy was defined as the prescription of five or more different medications in one year [9]. The first four characters of the ATC codes (ATC-level 3) were used to count medication prescriptions.

### Statistical analysis

Age and sex stratified analyses were used to assess the multimorbidity and polypharmacy trajectories of the study population and the subgroups. To analyze trajectories for the young as well as the older age groups, we categorized age into four categories: 0–24 years, 25–44 years, 45–64 years, 65 years and older at baseline in 2000 [35]. A discrete-time likelihood model—following Allison (2014)–was used to analyze the clinical trajectories during the fifteen-year follow-up period [44]. This model implements a time-to-event survival analysis, both for the time to the first event and for repeated events and was used to evaluate incident multimorbidity. A generalized estimating equations model (GEE), which included robust variance estimation as well as a likelihood-based generalized linear mixed model (GLMM) with multilevel random intercept specification to account for the correlation of longitudinal data within individuals was used to analyze the occurrence of repeated events; between-subjects variance following Heck et al. (2012, 2014) [45, 46] was measured to estimate how much variance in multimorbidity exists between individuals when correlated time data is accounted for. Trends over a specific period were described in terms of the proportion of people with multimorbidity and polypharmacy whereby the dependent variable was included as a binary variable [46]. Since the key variables age, sex and the incidence of chronic health conditions are strongly interrelated, adjustment for age and sex is clinically relevant. In this study we therefore used stratification by age and sex and presence or absence of multimorbidity at baseline, and/or covariate adjustment. For covariate adjustment, we adjusted for the variables age, sex and presence of the number of chronic conditions at baseline by explicitly including them in the statistical model. The statistical models—logistic regression, a generalized estimating equations (GEE) and multilevel analysis—were used to study the effect of the possible predictors, starting with the separate analysis of each variable, followed by multivariate analysis taking into account the effect of these predictors, and subsequently by adding interaction terms [8, 46].

In addition, trends over a specific period of time or over the whole period of the trajectory were described by using relative change whereby the relative change between two values of a variable was expressed as a ratio. Prevalence or (cumulative) incidence ratios thus indicate a relative increase or decrease in prevalence or incidence over the study period. Ratios were calculated between the first and last years of a trajectory.

For specific age and sex related subgroups odds and odds-ratios were estimated. These estimates were converted to predicted probabilities in the form of population or subgroup average [46]. Hierarchical age-period-cohort (HAPC) models [47, 48] were used to generate age and cohort trajectories [47] for comparison with prevalence trends in multimorbidity literature. We used two-level and three-level random intercept models with period and cohort as random variables, whereby age was also added as a squared variable [47, 48]. A two-sided *p* value ≤ 0.05 was considered statistically significant. We conducted analyses of interactions between sex, age-group, baseline history of multimorbidity and time for the years of the 15-year trajectory. We tested models using time as both a discrete and a continuous variable. A two-sided *p* value ≤ 0.10 was considered statistically significant for the evaluation of the interaction terms. The models were performed using IBM SPSS Statistics software version 27. For the design and execution of this study we followed the guideline for observational studies, i.e. the Strengthening the Reporting of Observational Studies in Epidemiology (STROBE) checklist [49].

### Ethical approval

The study was conducted in compliance with Good Clinical Practice Guideline Procedures, the principles of the Declaration of Helsinki (version October 2008) and Dutch (Medical Research Involving Human Subjects Act and Personal Data Protection Act) law. The privacy regulation of the study was registered with the Dutch Data Protection Authority. According to Dutch legislation, neither obtaining informed consent nor approval by a medical ethics committee is obligatory for observational studies.

## Results

### Population characteristics at baseline

The study population numbered 10037 persons on January 1, 2000, henceforth referred to as (at) baseline. A complete dataset was available for the study population for the 15-year-period. The numbers and proportions of female and male subjects in the four age groups were evenly distributed, apart from the 65 years and over age group, which included 698 subjects (7.0%) (Table 1).

**Table 1:** Distribution of females and males in age groups in the study population (N = 10037).

*Age group (Year)*	*All*	*All-%*	*Female*	*Female-%*	*Male*	*Male-%*
*0–24*	2969	29.6	1553	29.7	1416	29.4
*25–44*	3369	33.6	1720	32.9	1649	34.3
*45–64*	3001	29.9	1510	28.9	1491	31.0
*65+*	698	7.0	443	8.5	255	5.3
*Total*	10037	100.0	5226	100.0	4811	100.0

The difference between the percentage of females (N = 5226; 52.1%) and males (N = 4811; 47.9%) was 4.2 percentage points (CI-95%: 2.2%–6.2%; p <0.001) and statistically significant. In the youngest and oldest groups, the proportion of females was higher than the proportion of males, while the proportion of males was higher in the 25–44 year and 45–64 year age groups (Table 1). These differences were not statistically significant.

Of the 10037 subjects, 6925 (69.0%) had no chronic health condition at baseline, while 19.5% had one chronic health condition, and 11.5% had multimorbidity. The proportion of females with multimorbidity was lower in the age groups 45–64 year and ≥65 years (17.6% and 40.6%) compared to males (21.3% and 48.4%), whereby the differences were 3.7% (confidence interval (CI)-95%: 0.96–6.64; p = 0.0086) and 7.8% (CI-95%: 0.16–15.38; p = 0.0455) respectively (Table 2).

**Table 2:** Distribution of chronic health conditions and medications at baseline, stratified by age and sex (%).

	*Females (%)*				*Males (%)*				*TOTAL (%)*				*Grand total (%)*
*Age group (yrs.)*	0–24	25–44	45–64	65+	0–24	25–44	45–64	65+	0–24	25–44	45–64	65+	
*Chronic conditions*													
*0*	88.0	72.6	53.0	33.9	89.4	76.7	51.4	25.1	88.7	74.6	52.2	30.7	69.0
*≥2*	1.5	6.5	17.6	40.6	1.8	6.2	21.3	48.2	1.6	6.3	19.4	43.4	11.5
*Medication*													
*0*	70.1	43.1	34.8	39.1	65.5	57.7	44.2	19.0	65.5	57.7	44.2	19.0	52.7
*≥5*	3.4	13.5	22.0	25.7	1.4	5.4	13.3	23.9	2.5	9.4	17.7	25.1	10.9
*Total N*	1553	1720	1510	443	1416	1649	1491	255	2969	3369	3001	698	10037
*Total %*	29.7	32.9	28.9	8.5	29.4	34.3	31.0	5.3	29.6	33.6	29.9	7.0	100

Overall, the prevalence of polypharmacy at baseline was 10.9% (Table 2). The overall prevalence for females (14.0%) was almost double that of males (7.6%). This sex difference was strongly age-related and particularly marked in the younger age groups 0–24 year (females: 3.4% vs males: 1.4%), 25–44 year (13.5% vs 5.4%) and 45–64 year (22.0% vs 13.3%). However, the trend converged for both sexes in the oldest age group, in which the proportion for males was closer to the proportion for females (23.9% vs 25.1%).

### Trends in multimorbidity over time: Evolution of incident multimorbidity and prevalence change

**Fig 1:**
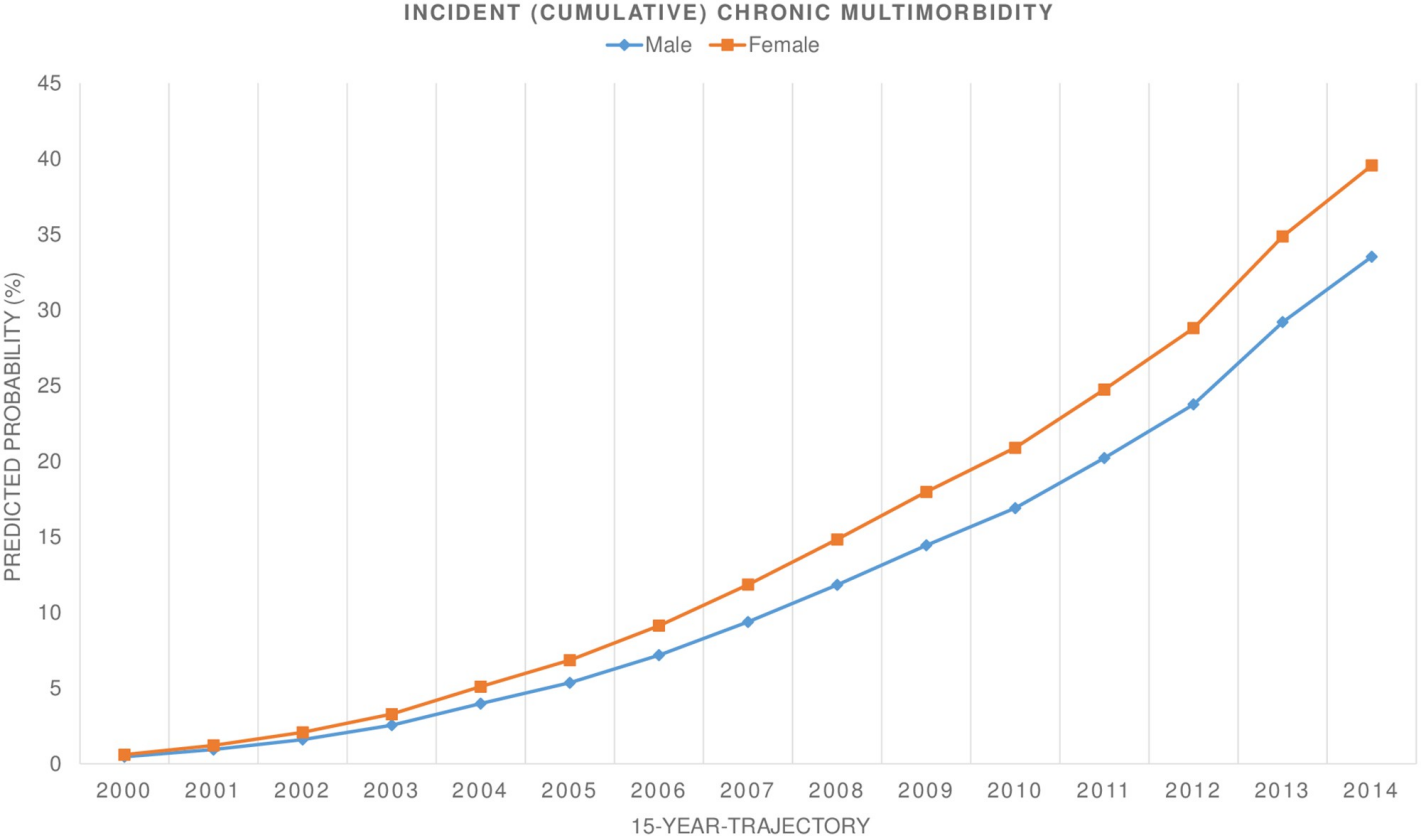
Incident (cumulative) multimorbidity (%) during 15-year trajectory stratified by sex.

The incident multimorbidity was shown to be higher in females than males over the 15-year period (Fig 1).

**Fig 2:**
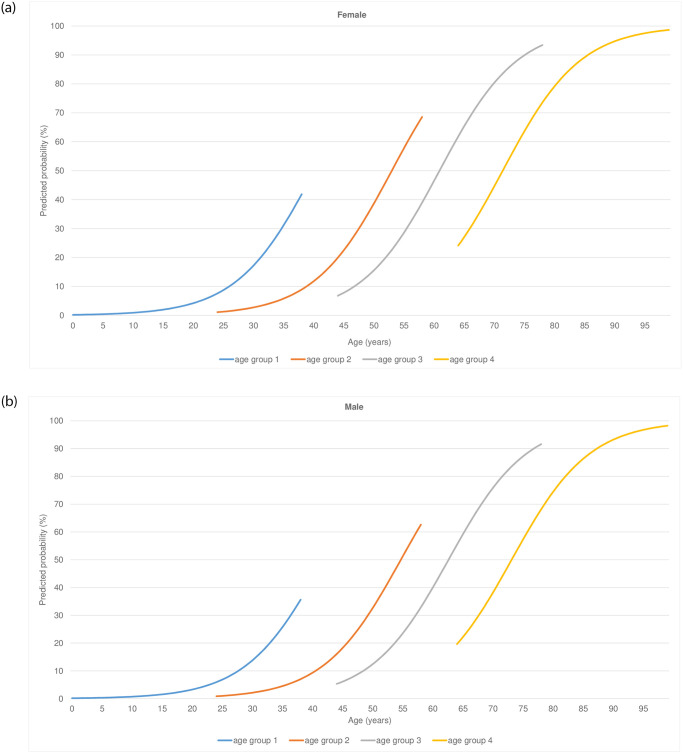
Age and age group effects on multimorbidity over time stratified by sex—Females (a) and males (b) (Y-axis: Predicted probability (proportion) of multimorbidity stratified for sex and age-groups (1 = 0–24 year; 2 = 25–44 year; 3 = 45–64 year; 4 = 65 year and older) during 15-year trajectory; X-axis: Age as time-varying variable, including a squared age-term).

In 2006, the proportion of females diagnosed with two or more chronic health conditions was about 2 percentage points higher than for males (9.2% for females versus 7.2% for males), with the gap widening to 39.6% for females and 33.5% for males by the end of the 15-year trajectory. The sex differences were also pronounced when stratifying sex-age subgroups (Fig 2).

Multimorbidity increased in all age-subgroups over the 15-year trajectory, but the rise was much more prominent in the younger female age groups (Fig 2). The estimated age effect was strong with a 16.7% higher likelihood of multimorbidity for each year of age as a continuous variable (OR = 1.17; CI-95%:1.16–1.18). The proportion of multimorbidity in the young female group– 0–24 years—was much higher at the end of the 15-year trajectory than in the comparable male group. Although the proportion of multimorbidity was somewhat higher in the oldest female group at the beginning of the 15-year trajectory, it ended the same as the oldest male group at the end. The 25–44 and 45–64 intermediate age groups showed a similar trend (Fig 2). In absolute percentage terms, the likelihood of multimorbidity was higher in the older age groups, but the relative increase in the younger age groups was stronger.

A similar pattern can be seen in the incidence rates. Whereas the overall incidence rate is 7.2 per 1000 person-years in the youngest age group, the incidence rate in the 0–24 female age group was 9.5 per 1000 person-years, which was about two times higher than the rate of 4.8 per 1000 person-years in the comparable male group. Conversely, the overall rate in the oldest age group was 78.6 per 1000 person-years and was almost similar in the oldest female and male groups, i.e., 78.9 and 77.8 per 1000 person-years respectively (see S2 Table).

Use of a two- and three-level random intercept model (mixed models—GLMM in SPSS) showed that the random variation between the years as periods was only 3.7%. Variance between subjects, which was the second level variable in the random intercept model, was estimated to be 0.552 suggesting that about 55.2% of the variance in incident multimorbidity lay between subjects. This likelihood varied significantly across individuals in the study (p < .001).

A comparison between the sex-age related evolution of multimorbidity with a cross-sectional trend analysis of prevalence of multimorbidity (measured as a crude incidence proportion), also showed that the relative increase of the incidence proportion was highest in the younger age-groups (Table 3).

**Table 3:** Prevalence in terms of the crude incidence proportion of multimorbidity (%) and its relative increase (ratio of prevalence in 2014 versus 2000).

*Gender-age group*	*Prevalence MM % 2000*	*Prevalence MM % 2014*	*Relative Increase MM* ^a^
*Female-0-24*	1.5	17.6	11.7
*Male-0-24*	1.8	10.7	5.9
*Female-25-44*	6.5	40.3	6.2
*Male-25-44*	6.2	34.2	5.5
*Female-45-64*	17.6	73.1	4.2
*Male-45-64*	21.3	73.0	3.4
*Female-65+*	40.6	90.7	2.2
*Male-65+*	48.2	92.2	1.9

^a^relative increase or relative change was calculated by computing the ratio between prevalence at the end of the follow-up period in 2014 and the beginning in 2000. For example, the relative increase in the prevalence of multimorbidity in the 0–24 female age group was 17.6/1.5 = 11.7.

At the end of the 15-year trajectory the prevalence of multimorbidity in the youngest female age group was 17.6% compared to 1.5% at the beginning and translating into a relative increase of 11.7 (Table 3). A similar trend could be seen in the youngest male group, albeit less prominently (relative increase: 5.9). In the oldest female group, the prevalence of multimorbidity at the end of the 15-year trajectory was 90.7%, compared to 40.6% at the beginning of the period. The increase was substantial, but the relative increase (2.2) was comparatively low. A similar trend can be seen in the male group (Table 3).

The results of the incidence proportions and the transformation in terms of prevalence in the closed cohort (N = 10037) above was also apparent in the cross-sectional analysis of an open and dynamic cohort taken from the RNFM database (see S3 Table).

**Fig 3:**
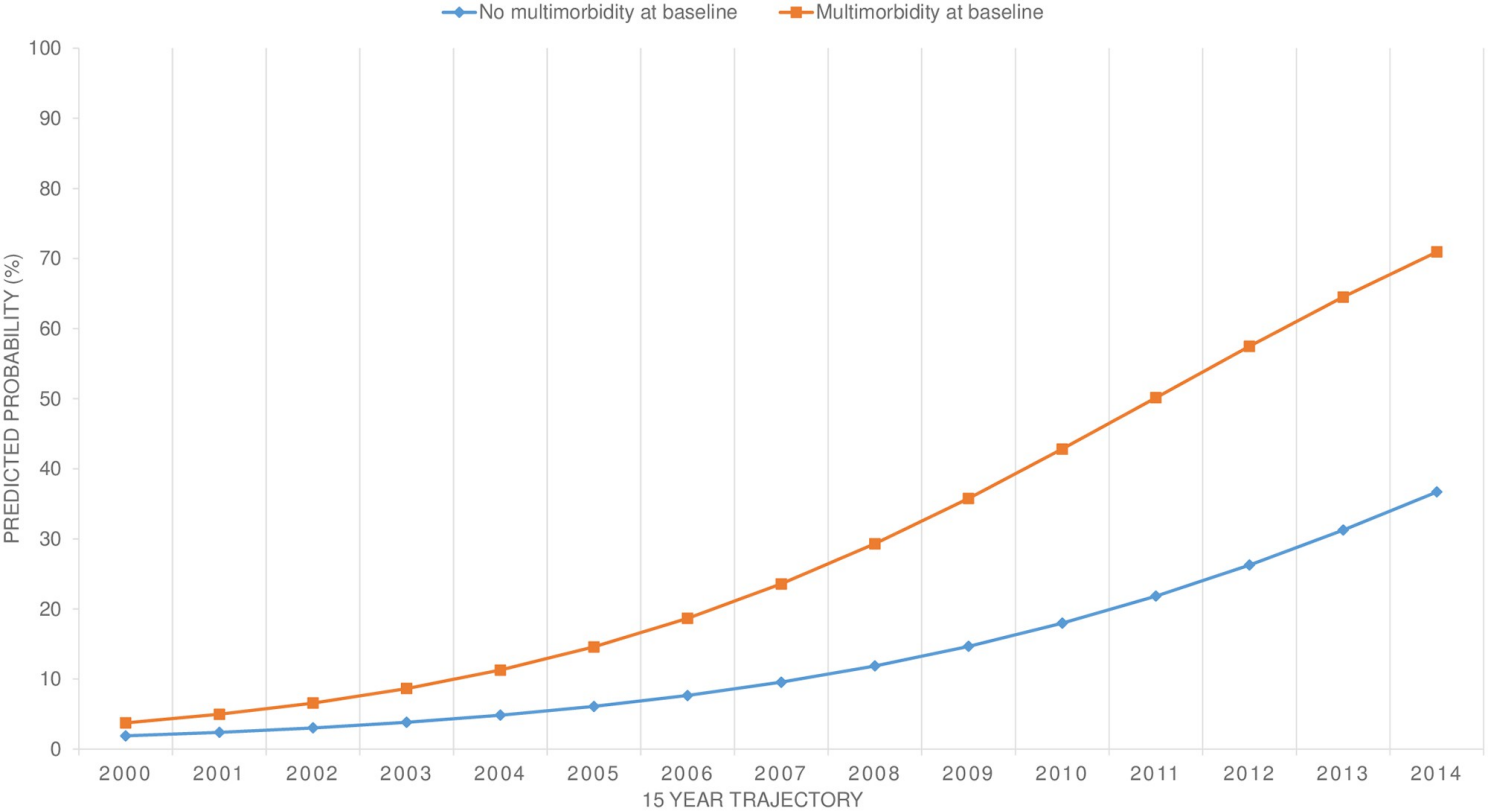
Predicted probability (cumulative) incident multimorbidity stratified according to medical history of multimorbidity at baseline.

The described trends were much stronger in patients who had multimorbidity in their medical history at baseline (Fig 3).

The cumulative incident multimorbidity during the 15-year trajectory was much higher in persons with two or more chronic health conditions at baseline than in those with no previous history of multimorbidity (Fig 3). The proportion of those diagnosed with two or more new chronic health conditions on top of their previous multimorbidity was 18.7% in 2006, or slightly under 20%. In subjects with no history of multimorbidity the 20% level was not breached until 2011, or about 5 years later when it was 21.8%. At the end of the 15-year trajectory, the difference between the two subgroups of persons with and without a previous history of multimorbidity had grown to more than 30% (70.9% and 36.7% respectively).

Since interaction analysis showed that the presence of multimorbidity at baseline was likely to be an effect modifier, the multivariate analyses were also stratified for the presence and absence of multimorbidity at baseline [8]. In addition, stratification by sex and age was performed next to using these variables together with interaction terms in the statistical analyses [36]. Significant interactions were found between sex and age-group (p = 0.019) or sex and age (p = 0.001), and baseline history of multimorbidity with age-group (p = 0.055), multimorbidity at baseline and time (p = < .001), and sex and time (p = 0.016).

### Trend in polypharmacy

**Fig 4:**
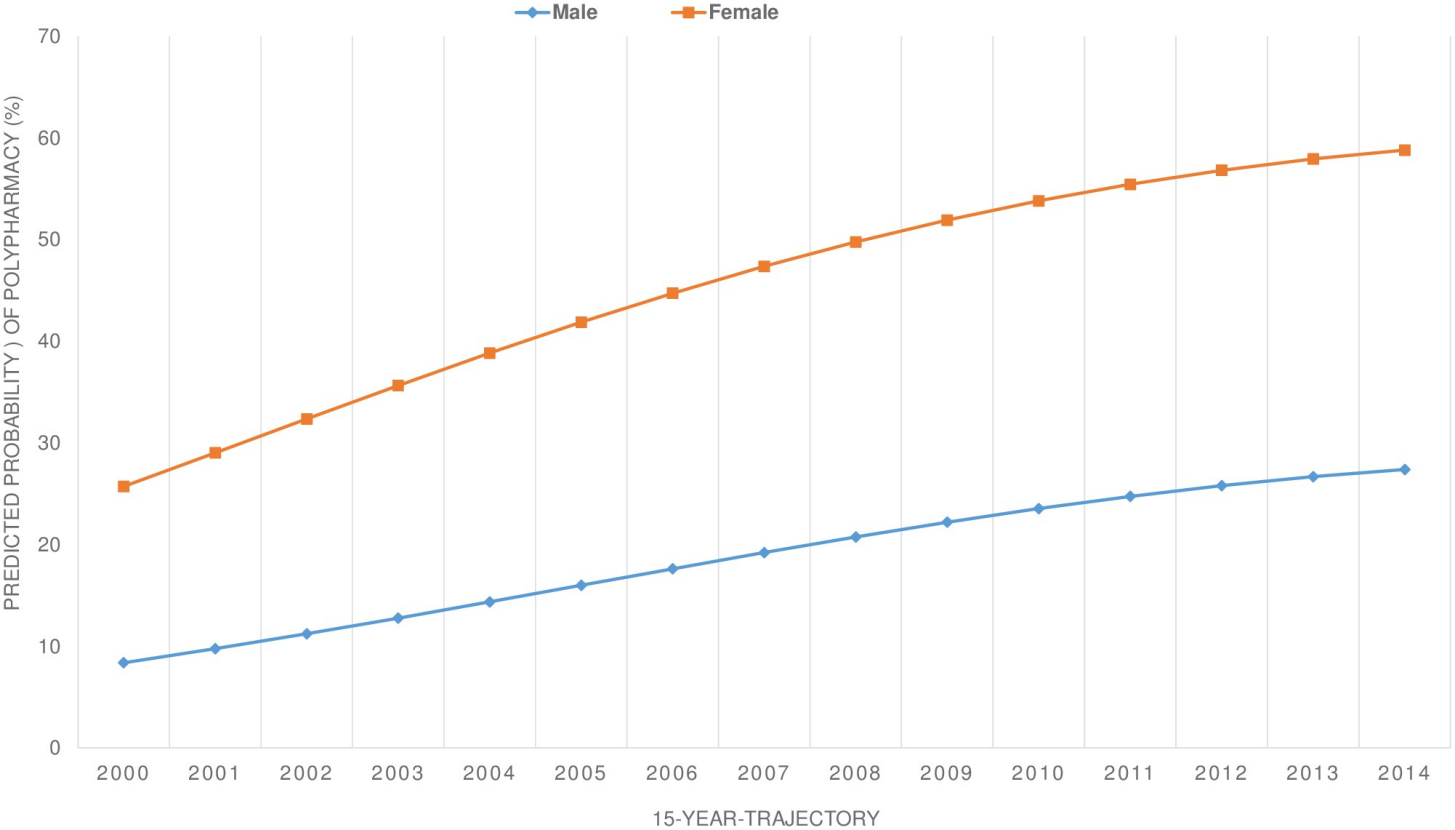
Predicted cumulative proportion of polypharmacy over the 15-year trajectory stratified by sex.

Based on a generalized estimating equation model with time, the square of time, and sex as interaction terms, the predicted probability of polypharmacy was much higher for females than for males (Fig 4). The proportion of females with polypharmacy was 25.7% in 2000, or about three times as high as for males (8.4%). This sex gap increases from 17.3 percentage points at baseline to over 30 percentage points at the end of the 15-year trajectory (58.8% for females; 27.4% for males) (Fig 4). The variance between subjects (as a second level variable in a random intercept model) was estimated to be 0.542, implying that the between-subject variance in polypharmacy was about 55%. The probability varied significantly across individuals in the study (p < .001).

**Fig 5:**
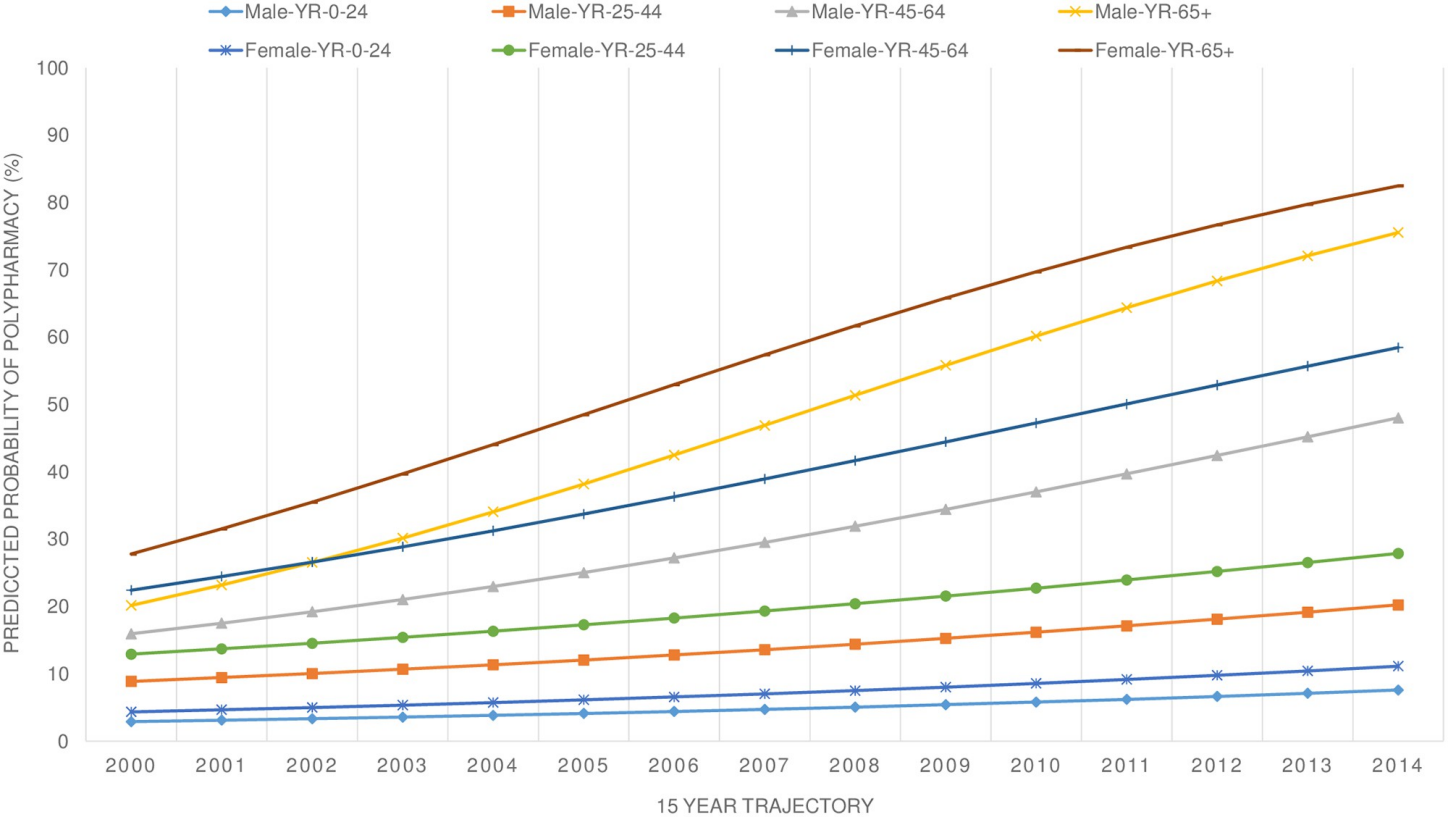
Predicted cumulative proportion of polypharmacy during 15-year trajectory stratified by sex and age.

This sex-related difference was also strong in each of the sex-age subgroups (Fig 5).

Polypharmacy increased steadily over the 15-year period in all age-subgroups. The young female group– 0–24 years—ended the 15-year trajectory much higher than the male young group, whereas the oldest male group started even lower than the 45–64 female group but ended in a similar fashion as the oldest female group. The intermediate age groups showed a similar trend. The odds ratio of polypharmacy–taking the youngest group of 0–24 years as a reference—was 3.2 (CI-95%: 2.8–3.6) in the 25–44 age-group, 10.7 (CI-95%: 9.5–12.0) in the 45–64 age-group, and 21.1 (CI-95%:18.2–24.4) in the 65 years and older group. When incident multimorbidity was included in the model, at both baseline and over time, the odds ratio in the 25–44 group dropped to 2.6 (CI-95%:2.3–2.9), in the 45–64 age-group to 5.9 (CI-95%: 5.3–6.5) and in the 65 years and older group to 7.9 (CI-95%:6.8–9.2). The changes in the odds ratios were thus substantial with the odds ratio of polypharmacy in the oldest group in comparison with the youngest group falling from to 7.9 from 21.1, when the presence or emergence of multimorbidity was accounted for. This becomes even more significant in view of the fact that sex, age, multimorbidity at baseline and incident multimorbidity over the 15-year period, also showed highly significant interactive associations (p<0.001).

In order to compare the sex-age related evolution of polypharmacy with a cross-sectional trend analysis of prevalence of polypharmacy, the following table is illustrative and shows that the relative increase of the incidence proportion was highest in the younger age-groups (see Table 4). The table shows that the relative increase was inversely proportional to age, with the relative increase in polypharmacy prevalence in females somewhat lower than in the comparable male age groups. However, with the exception of the oldest group, the female age-groups started at a higher level than the male groups.

**Table 4:** Comparison of the prevalence (%) of polypharmacy (PP) at the start and the end of the 15-year trajectory in the fixed cohort in absolute terms and relatively.

	*Prevalence PP 2000 (%)*	*Prevalence PP 2014 (%)*	*Relative Increase (prevalence ratio)*
*Female*			
*0–24*	2.3	11.1	4.8
*25–44*	6.6	26.9	4.1
*45–64*	16.8	51.4	3.1
*65+*	23.3	61.4	2.6
*Male*			
*0–24*	1.5	7.3	4.9
*25–44*	4.3	19.1	4.4
*45–64*	11.4	40.3	3.5
*65+*	16.2	50.4	3.1

Comparing the prevalence of polypharmacy in 2000 versus 2014 according to sex and age, the relative increase was again highest for the younger age groups, with a somewhat higher relative increase in the male age groups from the age-group of 25–44 years and above. It should be noted that the relative increase for the younger age groups was much more moderate for polypharmacy than for multimorbidity.

**Fig 6:**
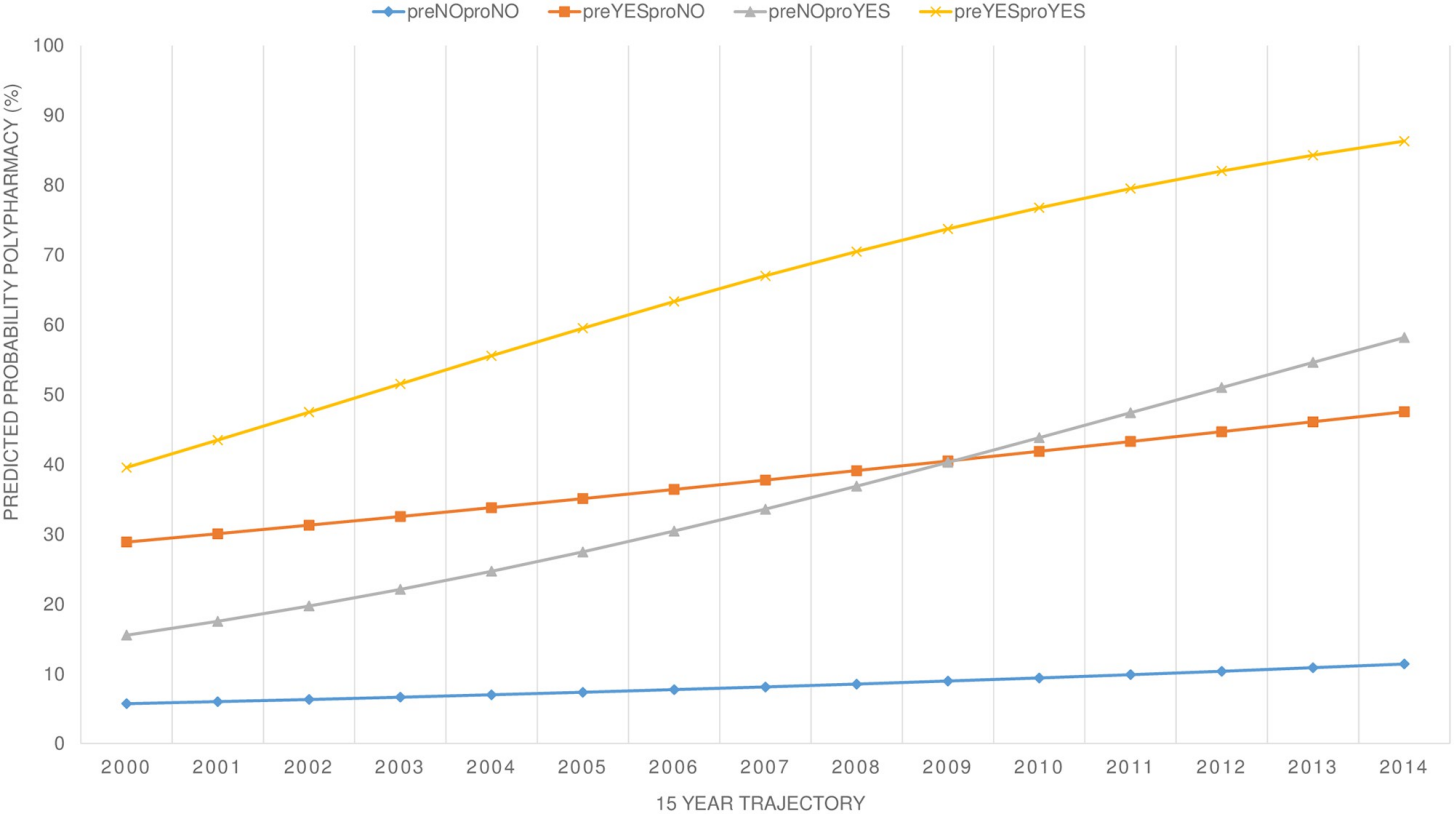
Predicted probability of polypharmacy stratified by presence of multimorbidity at baseline (pre) in combination with emergence of incident multimorbidity during 15-year trajectory (pro).

The described trends were much stronger in patients that had multimorbidity at baseline (Fig 6). These patterns were made visible by distinguishing between four subgroups: those with and without multimorbidity at baseline and those with or without developing incident multimorbidity during the 15-year trajectory (Fig 6).

The proportion of polypharmacy in persons that did not have multimorbidity in their medical history before the year 2000 and did not develop multimorbidity started at 5.7% in 2000 and ended at 11.4% in 2014. In those without multimorbidity at baseline but developing incident multimorbidity, these proportions rose to 15.5% and 58.2% respectively. For the individuals that had multimorbidity in their medical history but did not develop incident multimorbidity, the proportions of polypharmacy were 28.9% at the beginning and 47.5% at the end of the 15-year trajectory. Among those that had multimorbidity at baseline and developed further multimorbidity, the proportions of polypharmacy were 39.5% at the beginning and 86.3% at the end of the period. This difference in the polypharmacy trend is reflected in an odds ratio for multimorbidity at baseline of 1.47 (CI-95%: 1.26–1.70) and for multimorbidity over the 15-year period as a time-varying variable 3.78 (CI-95%: 3.40–4.19).

The interaction terms between previous and incident multimorbidity, age groups and time are substantial and statistically significant. Although estimated probabilities and odds ratios for polypharmacy did change depending on which model used—e.g., generalized estimating equations (GEE), random intercept model, or age-period-cohort model -, the trends as described were similar. This was the case regardless of whether time was a discrete or continuous variable in the longitudinal analysis (see S4 Table).

## Discussion

This study compared the 15-year trajectories of multimorbidity and polypharmacy stratified by sex and birth cohort groups. Our data showed that multimorbidity rose in line with age (i.e., prevalence in the oldest female was 90.7% and in the oldest male group 92.2%, or 5 and 9 time higher than in the youngest female (17.6%) and male (10.7%) age-groups (Table 3)). However, the younger age groups showed a relatively rapid increase in multimorbidity and, more moderately, in polypharmacy. The proportion of females with polypharmacy was much higher than that of males, and stood at 25.7% in 2000, compared with 8.4% in males. The 17.3 percentage point sex gap increased to over 30% at the end of the 15-year trajectory (58.8% for females; 27.4% for males). After stratifying according to sex-age subgroup, the differences continued to be pronounced in all groups. The results observed in the closed cohort were very similar to those found in the cross-sectional analysis of the open and dynamic cohort taken from the RNFM-database (see S3 Table). Although estimated probabilities and odds ratios for the various outcome measures changed (slightly), depending on which model was used (generalized estimating equations (GEE), random intercept model, or age-period-cohort model), the described trends were similar.

We sought to discover how multimorbidity progresses from an early age. As a result, our study adds to the literature on multimorbidity by identifying changes in the accumulation of chronic diseases and tracking differences in the patient trajectories across age and sex groups. Several aspects of our findings are noteworthy. Across a range of international settings, our findings largely agree with the results of other studies that multimorbidity prevalence increases with age [5, 9, 11, 24]. The prevalence in the youngest female and male groups was 1.5% and 1.8% respectively in the first year, rising to 17.6% and 10.7% respectively at the end of the 15-year period. This contrasts with the oldest female and male age groups, in which the prevalence started out at 40.6% and 48.2% and ended at 90.7% and 92.2% respectively. However, we found notable differences in the development of multimorbidity for the different subgroups. The relative increase in prevalence in the youngest female and male groups showed was 11.7 and 5.9 respectively, compared with 2.2 and 1.9 in the oldest female and male groups. In the 0–24 and 25-44-year age groups, we also found higher levels of multimorbidity among females and males, whereas the levels for converged in the older age groups, with a slightly higher prevalence of 92.2% for the males and 90.7% among females in the oldest group.

The early development of multimorbidity may indicate earlier and longer cumulative exposure to risk factors that are common to many of the chronic health conditions. Risk factors that assault multiple body systems, such as obesity, chronic stress, and systemic inflammatory episodes, may lead to earlier multimorbidity, as may other factors including depressive disorder, cognitive decline [8] and weaknesses in social networks and family support [8, 50] In addition, poor access to good quality health care and low SES may exacerbate and accelerate the development of multimorbidity [24, 47].

The few studies that have focused on chronic disease in relation to polypharmacy have generally been performed in specific chronic disease populations [10]. In the overall population, and particularly among older patient groups, chronic disease has been shown to be a significant risk factor for polypharmacy [9]. However, its prevalence is mostly assessed by repeatedly conducting cross-sectional polypharmacy studies. In a recent study, Maxwell compared the prevalence of polypharmacy in women and men aged 66 years and older in the year 2016 with that in 2003 [10]. In females, they found the prevalence had climbed only slightly from 64.2% in 2003 to 65.1% in 2016. In males, the prevalence rose from 55.7% to 62.3% over the same period, representing a relative increase of 1.1. Comparing prevalence proportions for six consecutive 2-year periods, Oktora et al. (2019) reported a relative increase of 2.5 for 2013–2014 versus 1999–2000 for the 25–44 and 45–64 age groups, and 2.1 for those aged 65 years and older [33]. Hence, as the authors note, the rise was steeper in the younger age groups than in the oldest age group [33]. We discovered sex-age ratios of 4.1, 3.1 and 2.6 in the female groups and 4.4, 3.5 and 3.1 in the male groups, confirming the trend towards a higher relative increase in the younger age groups (Table 4). The somewhat higher relative increases in the male groups indicate that in terms of prescription drugs, they were catching up on their female counterparts in all age groups.

Our findings confirm that polypharmacy is common among older patients and increases over time, as reported in various studies [9, 10, 29–33]. Age is an important factor driving the development of polypharmacy and the accumulation of chronic health conditions accelerates the use of multiple drugs. Particular attention should be paid to polypharmacy because it is associated with many drug-related problems such as adverse drug events, drug-drug-interactions, medication non-adherence, functional decline, and cognitive impairment [10, 24, 25, 33]. However, the clinical trajectory of a substantial portion of our patients accelerated and led to a further increase in polypharmacy across all age and sex groups. It is noteworthy that across all age and sex groups, including the youngest male and female groups, patients that developed multimorbidity early on, are at higher risk of a further episode of multimorbidity, along with an additional increase in the complexity of their therapies and medication regimens.

Two major trends were noticeable. The first trend was that young persons developed multimorbidity and polypharmacy much more rapidly than expected, even in comparison with the older individuals. The second trend was that a substantial group of patients continued to develop multimorbidity in the 15-year trajectories, increasing the complexity of their prescribed medication regimens. Strong interactive effects could also be observed and the increase in polypharmacy was most prominent among those with multimorbidity in their medical history and those that developed multimorbidity over the 15-year trajectory. This shows that polypharmacy and multimorbidity patterns may continue throughout the course of patients’ lives and include accelerating episodes. A substantial group of persons with incident multimorbidity also showed a similarly steep increase in polypharmacy. These two groups clearly differed from the other two groups which showed a more stable pattern of multimorbidity and polypharmacy.

### Context with previous findings

Although we calculated incidence rates, we did not aim to estimate them precisely for multimorbidity. Our study is to the best of our knowledge one of only a few that actually uses an individual-based patient trajectory analysis over a long time period to evaluate incident multimorbidity across age and sex groups. However, considerable variation in methodological choices and populations make incidence rates for chronic diseases, where available, difficult to compare [5, 9]. This also applies to reports on prevalence data. Nevertheless, some comparisons may be insightful. Maxwell et al. (2021) report that between 2003 and 2016 –the two index years in their study conducted in a US population setting—the prevalence of multimorbidity among persons aged 66 years and older increased from 75.9% to 82.1% in women and 72.5% to 79.4% in men [10]. The authors attributed the difference between the prevalence in 2003 and 2016 to a general increase in the risk of multimorbidity. At the end of the 2000–2014 period, we found a prevalence of 90.7% in women and 92.2% in men aged 65 years and older. The higher prevalence may be partly explained by an increase in the individual multimorbidity trajectories of women and men. However, Maxwell et al. (2021) noted that higher estimates of prevalence in the same US setting were associated with the inclusion of a lower number of chronic conditions [10]. They noted that other studies incorporated about 20 chronic conditions to assess the state of multimorbidity, whereas the study by Maxwell included 31 chronic conditions [10]. Our study used the full range of 88 chronic conditions in the primary care setting.

For the youngest age group, the available literature is very limited. Bjur et al. (2019) compared the incidence and prevalence of multimorbidity within the 0–17 age group over three consecutive 5-year periods (with the index years 1999, 2004 and 2009 for incidence and 2004, 2009 and 2014 for prevalence) [25]. The incidence rates were 2.5, 2.7 and 3.3 per 1000 person-years respectively, compared with the higher rate of 7.2 per 1000 person-years that we found. Similarly, the 12% prevalence proportion that we found in 2014 was higher than those of Bjur at 1.2%, 1.5% and 2.0% in the three index years. However, Bjur et al. based their analysis on 31 conditions [25]. St Sauver et al. (2015) included 20 chronic conditions and—measuring only incidence rates—found an incidence rate over a 14-year period of 6.5 per 1000 person-years for a 0–19 age groups and 260.0 per 1000 person-years for the aged 80 years and older [11]. Our rates were 7.4 for the 0–24 age group and 78.6 in the group aged 65 years and older. St Sauver found comparable incidence rates for women and men with overall incidence rates of 38.8 and 35.5 per 1000 person-years respectively. Interestingly, the authors studied in detail developments in rates for both the chronic conditions under review and co-morbid conditions and found very different combinations in females and males with highly different rates [11]. We also found a higher rate for females than for males. Moreover, we found a marked difference between sex-age groups. For example, in the 0–24 age groups, the incidence rate per 1000 person-years was 9.5 for females and 4.8 for males. For the oldest groups of 65 years and older, the sex-rates were similar at 78.9 and 77.8 per 1000 person-years for females and males respectively (see S2 Table).

We also converted the temporal trends in multimorbidity and polypharmacy to prevalence trends for both outcome measures in order to compare them with prevalence findings in the literature. The prevalence patterns in age and sex groups in our study indicated similar trends to those in other studies. For the Dutch population between 1995–2000, Uijen et al. found a modest increase of people with two or three chronic conditions, and a more pronounced increase in people with four or more chronic diseases [51]. Even more pronounced results were reported by a recent study using primary care data in Belgium [9]. These results agree with our findings of a much steeper increase in incident multimorbidity in those with multimorbidity in their medical history at baseline. However, our results on the evolution of multimorbidity may indicate that such accelerating patterns occur at various phases in the course of patients’ lives. Van Oostrom et al. showed a modest standardized increase of 2.7% over a 7-year period for people aged 75 years and older [52], while Meinow et al. found stable prevalence proportions of multiple severe symptoms/diseases among older people (over 77 years of age) between 2002 and 2011 [53]. These findings were based on self-reports of chronic health conditions. In a study extending from 1994–2011, Canizaris et al. showed that more recent birth cohorts had a higher risk of developing multimorbidity, but also obtained these results from self-reports [47]. Our results showed somewhat higher prevalence proportions, but more importantly, we found much more dynamic patterns in prevalence, with a marked relative increase in all age groups, and most prominently in the youngest age groups. This agrees with a study by Van den Akker et al. (2019), in which the authors used a cross-sectional period design with joinpoint regression analysis and observed also arising prevalence in all age groups, with a higher rate of increase in the younger age groups. The authors speculated that this may reflect an increase in the efficiency with which health conditions in the younger groups are coded [9]. This may still hold true, but our study also showed that a renewed pattern of incident multimorbidity amongst those that had previously developed multimorbidity existed in the older age groups. We also found a strong age effect as well as age-group effects in the course of patients’ lives. This is similar to the findings of Canizaris et al. (2018) who used an age-period-cohort design and showed a marked relative increase in the younger age groups in addition to a strong overall age effect [47].

Several authors have suggested that the higher reported prevalence of chronic conditions is likely related to changes in diagnostic and treatment practices over time and does not simply reflect worsening health in the overall population [5, 9, 47]. However, Canizaris et al. (2018) suggested that multimorbidity is not only becoming the norm but emerging earlier in people’s lives, particularly in obese individuals and those with a lower income [47]. In addition, such sociodemographic factors as social deprivation and ethnicity, smoking etc. may influence the order in which chronic health conditions develop [17]. This highlights the relevance of understanding the influence that different combinations of individual factors and contextual factors can have on health trajectories. It also indicates that new patterns of multimorbidity may emerge in future cohorts as they age [47]. Putting aside the debate on whether recent generations are healthier or not than the previous ones [5, 9, 47, 54], it should be noted that age-cohort-period models control for variability across periods of time, but do not themselves estimate period trends. More importantly, they do not analyze incident morbidity patterns in a longitudinal design as was the case in this study. Focusing on trajectories may be the key to obtaining meaningful in-depth analysis in the future. Our study supports the findings of Vetrano et al. that the clinical trajectories of older adults with multimorbidity are characterized by great dynamism and complexity but can still be tracked over time [5]. However, such dynamism and complexity was also found in the young sex-age subgroups, and we discovered accelerating episodes of multimorbidity in the various phases of such patient’s lives. Young age groups thus also show dynamic and complex patterns of multimorbidity and polypharmacy.

### Strengths and limitations

The analyses for this study were performed using a closed cohort selected from the large RNFM database and the consequent availability of information on disease status increased the internal validity of our study. We compared the presented patterns and trends with the information we already had on about 80,000 active patients and found similar results, which supported the external validity. The RNFM database represents the population in the South of the Netherlands, which is not representative for the overall Dutch general population, but is nevertheless comparable to it. For comparison with other studies in the literature, we stratified the analyses by sex and age. We described the trends and tested various models by using age and time both as categorical and continuous variables. We also performed different types of longitudinal analyses using, e.g., a generalized estimating equation, random intercept, and age-period-cohort models. Although the estimated parameters, i.e. odds and odds-ratio’s differed, the described trends were comparable (see S2 Table). A major strength of this study is the use of longitudinal models to account for the correlated data and enable incident multimorbidity to be analyzed over a long time period of 15-years, and its relationship with polypharmacy trajectories to be tracked. In addition to the long investigation period, the large span in the age of the participants, and the inclusion of young and very young age groups is a strength of the study.

An additional strength of this study is the analysis of a large, high-quality electronic health record database, whose reliability and completeness has already been established [cf. 1, 9, 18, 37, 40]. It is important to stress that in the Netherlands, the GPs have comprehensive information on the health status of their patients because GPs are gatekeepers to specialist health care facilities. It is also compulsory for Dutch residents to have health care insurance and to register with a GP. Furthermore, clinical specialists in the hospitals and other health care institutions routinely provide GPs with information on the diagnosis, treatment and care of their patients.

Several limitations of the present study should also be mentioned. First, although external validation of primary care databases such as the RNFM database has been performed by means of national and international comparisons, previous analyses have shown that the registration of medication is not always complete [9]. This may result in an underestimation of polypharmacy rates, but we do not expect this to have affected the reported trends. Second, a strict definition of multimorbidity and polypharmacy was used, i.e. the occurrence of two or more chronic conditions and five or more different medication prescriptions in one year. This definition of polypharmacy may be too strict. In paediatrics for example, polypharmacy for children is defined as the prescription of two or more medications [27]. Our youngest group of 0–24 years included children, but we did not distinguish between them and other group members. Nevertheless, our data showed that the age effect in this subgroup was prominent, both for the females and males, and both in terms of incident multimorbidity and for the prevalence trend of polypharmacy. Although we evaluated the incidence of chronic diseases over the fifteen-year-trajectories, this was not the case for the prescription of medications. This is because it is not possible to match the prescription medication with the condition for which they were prescribed. Third, diseases were considered regardless of their severity. Disease severity may partially explain the clinical trajectories described in the present study. However, the interactions between various chronic diseases may play a major role as has been shown in previous studies [5]. Overall, the insights into the evolution of multimorbidity and polypharmacy provided in this study are most valuable and fill an important knowledge gap that cannot be filled by cross-sectional studies.

### Implications for clinical practice and research

Over the course of their lives, many individuals develop multiple chronic health conditions. As health care professionals and the health care and services system must face the challenge of dealing with these conditions, it is important to investigate the clinical trajectories of individual patients by moving beyond a single disease approach and focusing on the dynamics and complexities of multimorbidity and polypharmacy. The analyzed patterns are interesting from the perspective of daily care provided by GPs [18]. To the best of our knowledge, this study is one of few to investigate the dynamics of multimorbidity and polypharmacy in primary care from a longitudinal perspective. Considering that the diagnoses concern only the registered chronic health conditions and leave out symptoms and signs, medical procedures and other health care activities, it is evident that complex care is a challenge for the GP. Given GPs’ expertise in dealing with multimorbidity and their overview of the course of patients’ lives, GPs require insight into multimorbidity and polypharmacy patterns. Hence, primary care and clinical health care are in urgent need of more information on the clinical trajectories and evolution of chronic health problems and repeat medical prescriptions in patients’ lives. This underlines the need for care innovation in this group of complex patients, including consideration of patient’s preferences [19]. Various approaches meet the needs of patients such as the method of minimally disruptive medicine [9, 19] and the use of the Ariadne principles [9], which offer guidance on how to manage multimorbidity in primary care consultations [5, 9, 19, 20]. These and other approaches acknowledge the importance of the patient’s role and the patient-physician communication in health care. GPs need to understand that their practice contains groups of patients with different multimorbidity and polypharmacy patterns. A substantial number of patients develop multimorbidity in early life and develop further health problems over their lives. Accelerating phases of multimorbidity and the number of medicines patients take may signal critical stages in their clinical trajectories. Epidemiological results help better understand the development of multimorbidity and polypharmacy by highlighting reasons for variations in patient outcomes and enabling targeted interventions to be designed to manage adverse outcomes of multimorbidity. Establishing the incidence of multimorbidity and polypharmacy is a critical step in understanding the etiology of multimorbidity and helping to prevent its development in general and specific patient populations throughout their lives. Furthermore, it will help support the identification of high-risk patient groups and the implementation of preventive measures.

Multimorbidity and polypharmacy studies are particularly lacking in children and adolescents. However, the incidence of multimorbidity among children and adolescents warrants more attention given their increased risk of developing additional diseases later in life.

The epidemiology of chronic disease, multimorbidity and polypharmacy is dynamic and might differ according to region. Since the disease and medication profiles of patient groups in primary and secondary care are very different, it is important to make use of primary care databases—apart from databases containing on stays in hospital—to describe multimorbidity and polypharmacy in the general population. Reliable and up-to-date analyses are necessary to guide health policy, physicians and in the development of medical guidelines. Examples of the application of epidemiological findings can be found in two recently published national guidelines for polypharmacy [55, 56].

Differences in the trends in specific age and sex-groups reinforce the need for further research to substantiate these findings and to further distinguish between different trajectories in order to advance our understanding of the evolution of multimorbidity and polypharmacy over patients’ lives.

## Conclusion

For all sex-age groups, multimorbidity and polypharmacy are frequent, dynamic and intensify over time. In addition, younger sex-age groups are exhibiting a relatively rapid increase, whereby subsets of all the sex-age groups show accelerating trends in multimorbidity with accompanying polypharmacy. Multimorbidity and polypharmacy research faces many challenges, including the implementation of a longitudinal, life course approach from a family medicine perspective.

## Supporting information

S1 Table ICPC codes of 88 chronic diseases. (DOCX)

S2 Table Multimorbidity incidence rates stratified for sex, age and multimorbidity at baseline. (DOCX)

S3 Table Comparison prevalence of multimorbidity in fixed cohort N = 10037 and a cross-section of the open and dynamic RNFM cohort (N = 69,953). (DOCX)

S4 Table Odds ratio estimates for polypharmacy in two types of models, using time as both a continuous and a discrete variable. (DOCX)
