# Supplementary material for: Fifteen-year trajectories of multimorbidity and polypharmacy in Dutch primary care—A longitudinal analysis of age and sex patterns
Source: PLoS One. 2022 Feb 25;17(2):e0264343. doi: 10.1371/journal.pone.0264343 (PMC8880753; doi:10.1371/journal.pone.0264343)
Supplement: S1 Table — (DOCX) [file pone.0264343.s001.docx]

S1 Table. ICPC codes of 88 chronic diseases

| **ICPC-code** | **Title** |
| --- | --- |
| A79 | Carcinomatosis (unknown primary site) |
| A90 | Multiple cong anomalies |
| B72 | Hodgkin’s disease |
| B73 | Leukaemia |
| B74 | Other malignant neoplasms |
| B78 | Hereditary haemolytic anaemia |
| B83 | Purpura/coagulation defects/abnormal plate |
| B90 | HIV-infection (AIDS/ARC) |
| D74 | Malignant neoplasm stomach |
| D75 | Malignant neoplasm colon/rectum |
| D76 | Malignant neoplasm pancreas |
| D77 | Malignant neoplasm other/unspecified |
| F83 | Retinopathy |
| F84 | Macular degeneration |
| F94 | Blindness all degrees/types |
| H83 | Otosclerosis |
| H86 | Deafness all degrees NOS |
| K74 | Angina pectoris |
| K76 | Other/chronic ischemic heart disease |
| K77 | Heart failure |
| K82 | Pulmonary heart disease |
| K86 | Uncomplicated hypertension |
| K87 | Hypertension with involvement of target organs |
| K90 | Stroke/cerebrovascular accident |
| K91 | Atherosclerosis excl heart/brain |
| K92 | Other arterial obstruction/peripheral vascular disease |
| K93 | Pulmonary embolism |
| K94 | Phlebitis/thrombophlebitis |
| L84 | Osteoarthritis of spine |
| L85 | Acquired deformities of spine |
| L88 | Rheumatoid arthritis/allied conditions |
| L89 | Osteoarthritis of hip |
| L90 | Osteoarthritis of knee |
| L91 | Other osteoarthritis |
| L95 | Osteoporosis |
| L98 | Acquired deformities of limbs |
| N70 | Poliomyelitis/other enterovirus |
| N74 | Malignant neoplasms |
| N85 | Congenital anomalies |
| N86 | Multiple sclerosis |
| N87 | Parkinsonism |
| N88 | Epilepsy all types |
| N89 | Migraine |
| N90 | Cluster headache |
| N92 | Trigeminus neuralgia |
| P70 | Dementia senile/Alzheimer |
| P71 | Other organic psychosis |
| P72 | Schizophrenia all types |
| P73 | Affective psychosis |
| P74 | Anxiety disorder/anxiety state |
| P75 | Hysterical/hypochondriacal disease |
| P76 | Depressive disorder |
| P77 | Suicide attempt |
| P79 | Other neurotic disorder |
| P80 | Personality disorder |
| P85 | Mental retardation |
| P98 | Other/unspecified psychosis |
| R84 | Malignant neoplasm bronchus/lung |
| R85 | Other malignant neoplasm |
| R91 | Chronic bronchitis/bronchiectasis |
| R95 | Emphysema/COPD |
| R96 | Asthma |
| S77 | Malignant neoplasms of skin |
| S87 | Atopic dermatitis/eczema |
| S91 | Psoriasis |
| S97 | Chronic ulcer skin (incl varicose) |
| T71 | Malignant neoplasm thyroid |
| T80 | Other congenital anomalies |
| T85 | Hyperthyroidism/thyrotoxicosis |
| T86 | Hypothyroidism/myxedema |
| T88 | Renal glucosuria |
| T90 | Diabetes mellitus |
| T92 | Gout |
| T93 | Lipid metabolism disorder |
| T99 | Other endocrine metabolic nutritional disease |
| U75 | Malignant neoplasm of kidney |
| U76 | Malignant neoplasm of bladder |
| U77 | Other malignant neoplasm urinary tract |
| U85 | Congenital anomalies urinary tract |
| U88 | Glomerulonephritis/nephrosis |
| W72 | Malignant neoplasm |
| W77 | Other non-obstetric conditions |
| X75 | Malignant neoplasm cervix |
| X76 | Malignant neoplasm breast |
| X77 | Other malignant neoplasms |
| Y77 | Malignant neoplasm prostate |
| Y78 | Other malignant neoplasm |
| Y85 | Benign prostatic hypertrophy |
