# Supplementary material for: Fifteen-year trajectories of multimorbidity and polypharmacy in Dutch primary care—A longitudinal analysis of age and sex patterns
Source: PLoS One. 2022 Feb 25;17(2):e0264343. doi: 10.1371/journal.pone.0264343 (PMC8880753; doi:10.1371/journal.pone.0264343)
Supplement: S2 Table — (DOCX) [file pone.0264343.s002.docx]

S2 Table. Multimorbidity incidence rates stratified for sex, age and multimorbidity at baseline

| Multimorbidity (MM) Incidence Rate (IR) per 1000 person-years of 15-year trajectory | | | | | | | |  |
| --- | --- | --- | --- | --- | --- | --- | --- | --- |
| **A.** No multimorbidity at baseline (N=8887) | | | | | | | |  |
|  | Overall | | Female | | Male | | |  |
|  | 24.5 | | 27.0 | | 21.9 | | |  |
| Age group (years) |  | |  | |  | | |  |
| 0 – 24 | 7.2 | | 9.5 | | 4.8 | | |  |
| 25 – 44 | 20.2 | | 22.2 | | 18.1 | | |  |
| 45 – 64 | 49.3 | | 50.8 | | 47.6 | | |  |
| 65+ | 78.6 | | 78.9 | | 77.8 | | |  |
| **B.** Multimorbidity at baseline (N=1150) | | | | | | | | |
|  | | 58.9 | | 60.4 | | 57.3 | | |
| Age group (years) | |  | |  | |  | | |
| 0 – 24 | | 8.5 | | 12.6 | | 5.2 | | |
| 25 – 44 | | 27.3 | | 27.4 | | 27.2 | | |
| 45 – 64 | | 66.1 | | 69.4 | | 63.5 | | |
| 65+ | | 84.9 | | 81.6 | | 89.8 | | |
| **C.** Total (N=10037) | | | | | | |  |  |
|  | | 27.9 | 30.2 | | | 25.5 |  |  |
| Age group (years) | |  |  | | |  |  |  |
| 0 – 24 | | 7.3 | 9.6 | | | 4.8 |  |  |
| 25 – 44 | | 20.6 | 22.6 | | | 18.6 |  |  |
| 45 – 64 | | 52.4 | 53.9 | | | 50.8 |  |  |
| 65+ | | 81.3 | 80.0 | | | 83.5 |  |  |
