# Supplementary material for: Fifteen-year trajectories of multimorbidity and polypharmacy in Dutch primary care—A longitudinal analysis of age and sex patterns
Source: PLoS One. 2022 Feb 25;17(2):e0264343. doi: 10.1371/journal.pone.0264343 (PMC8880753; doi:10.1371/journal.pone.0264343)
Supplement: S3 Table — (DOCX) [file pone.0264343.s003.docx]

**S3 Table. Comparison prevalence of multimorbidity in fixed cohort N=10037 and a cross-section of the open and dynamic RNFM cohort (N=69,953)***

| Gender-age group | Prevalence MM  % 2000 | Prevalence MM  % 2014 | Relative Difference of Prevalence MM |
| --- | --- | --- | --- |
| Female-0-24 | 1.5 | 17.6 | 10.73 |
| Male-0-24 | 1.8 | 10.7 | 4.94 |
|  |  |  |  |
| Female-25-44 | 6.5 | 40.3 | 5.20 |
| Male-25-44 | 6.2 | 34.2 | 4.52 |
|  |  |  |  |
| Female-45-64 | 17.6 | 73.1 | 3.15 |
| Male-45-64 | 21.3 | 73.0 | 2.43 |
|  |  |  |  |
| Female-65+ | 40.6 | 90.7 | 1.23 |
| Male-65+ | 48.2 | 92.2 | 0.91 |

*Prevalence of multimorbidity (%) and relative difference (as a ratio: prevalence in 2014 divided by prevalence 2000)
