# Supplementary material for: Fifteen-year trajectories of multimorbidity and polypharmacy in Dutch primary care—A longitudinal analysis of age and sex patterns
Source: PLoS One. 2022 Feb 25;17(2):e0264343. doi: 10.1371/journal.pone.0264343 (PMC8880753; doi:10.1371/journal.pone.0264343)
Supplement: S4 Table — (DOCX) [file pone.0264343.s004.docx]

**S4 Table. Odds ratio estimates for polypharmacy in two types of models, using time as both a continuous and a discrete variable**

In the two models below we present the parameter estimates for four variables, i.e., sex, age as four age strata, presence or absence of multimorbidity at baseline (MM_at_baseline) and the incidence of multimorbidity (incident_MM) as a time-varying variable. The outcome is measured as polypharmacy present versus absent. The models include the time-related variable, i.e. time as a repeated measurement of 15 time points, i.e., the fifteen years of the trajectory. The first model uses time as a continuous variable where the second model uses time as a categorical variable. The estimates for the time variable are not shown here, since the objective here is to investigate the effect of the type of time-variable used on the estimates of the variables, that is the predictors of polypharmacy. The numbers represent estimated odds ratios. For example, the estimate for the OR=1.592 of the sex variable means that females (coded as 1) are about 1.6 times more likely to be in a state of polypharmacy than males (coded as 0). The CI-95% columns represent the 95%-confidence intervals of the estimated ORs. Three kinds of sub-models are evaluated, the first one including all four predictors, the second one including the first three predictors but not incident multimorbidity (MM), and the third sub-model only including sex and age as predictors.

Model 1 with time as continuous variable

| Variable | OR model all | CI-95% | OR model minus timevarying | CI-95% | OR model minus time varying and MM at baseline | CI-95% |
| --- | --- | --- | --- | --- | --- | --- |
| Sex | 1.592 | 1.484-1.709 | 1.575 | 1.461-1.698 | 1.479 | 1.372-1.595 |
| Age group 1 | 1 |  | 1 |  | 1 |  |
| Age group 2 | 2.593 | 2.327-2.889 | 3.055 | 2.709-3.445 | 3.183 | 2.816-3.598 |
| Age group 3 | 5.863 | 5.270-6.523 | 8.769 | 7.801-9.858 | 10.705 | 9.522-12.035 |
| Age group 4 | 7.888 | 6.781-9.175 | 12.908 | 11.034-15.099 | 21.059 | 18.194-24.376 |
|  |  |  |  |  |  |  |
| MM_at_baseline | 3.844 | 3.479-4.248 | 3.544 | 3.203-3.922 | X | X |
|  |  |  |  |  |  |  |
| Incident_MM as time-varying variable | 2.752 | 2.590-2.923 | X | x | X | x |

Model 2 with time as categorical variable

| Variable | OR model all | CI-95% | OR model minus timevarying | CI-95% | OR model minus time varying and MM at baseline | CI-95% |
| --- | --- | --- | --- | --- | --- | --- |
| Sex | 1.585 | 1.476-1.707 | 1.566 | 1.451-1.689 | 1.473 | 1.366-1.589 |
| Age group 1 | 1 |  | 1 |  | 1 |  |
| Age group 2 | 2.505 | 2.245-2.795 | 2.955 | 2.606-3.339 | 3.078 | 2.719-3.485 |
| Age group 3 | 5.571 | 5.002-6.205 | 8.478 | 7.532-9.542 | 10.318 | 9.168-11.614 |
| Age group 4 | 7.648 | 6.573-8.899 | 12.747 | 10.889-14.923 | 20.728 | 17.893-24.01 |
|  |  |  |  |  |  |  |
| MM_at_baseline | 3.865 | 3.497-4.278 | 3.544 | 3.202-3.921 | X | x |
|  |  |  |  |  |  |  |
| Incident_MM as time-varying variable | 2.863 | 2.694-3.043 | X | x | x | x |

We do not see substantial differences between the ORs as parameter estimates between the two models either using time-relating variable as a continuous variable or as a categorical variable. For example, the ORs for sex in the first model (OR=1.592) and the second model (OR=1.585) are very similar. The same holds true for the other parameters estimates.
